# The use of accelerometers to quantify external load in rugby league match play

**DOI:** 10.3389/fspor.2025.1560877

**Published:** 2025-03-24

**Authors:** Jemma A. Turner, Robert Delves, Heidi R. Compton, Lachlan Penfold, Grant M. Duthie

**Affiliations:** ^1^ High Performance Unit, Melbourne Storm Rugby League Club, Melbourne, VIC, Australia; ^2^ School of Exercise Science, Australian Catholic University, Strathfield, NSW, Australia; ^3^ Institue for Health and Sport (iHeS), Victoria University, Melbourne, VIC, Australia; ^4^ Applied Sport Science and Exercise Testing Laboratory, School of Biomedical Sciences and Pharmacy, University of Newcastle, Callaghan, NSW, Australia; ^5^ Sports Performance, Recovery, Injury and New Technologies (SPRINT) Research Centre, Australian Catholic University, Melbourne, VIC, Australia

**Keywords:** accelerometer, load, rugby, league, team, sport

## Abstract

**Introduction:**

This study quantified the distribution of accelerometer data in rugby league competition.

**Methods:**

A squad of 35 male professional National Rugby League (NRL) players (age: 26.0 ± 3.4 years; height 187.8 ± 6.4 cm; weight 98.7 ± 9.7 kg) wore inertial measurements units (Vector S7, firmware; 8.1, Catapult Sports, Victoria, Australia) during the 2023 NRL season. Three-dimensional 100 Hz acceleration data were exported and manually processed for each individual game file. An “acceleration index” was calculated by combining the three accelerometer signals, subtracting the influence of gravity, and removing periods of rest (5 s of less than 0.3 G). The “acceleration index” demonstrated a bimodal distribution of each individual player game file. A Gaussian mixture model was fitted to the acceleration index resulting in a mean, standard deviation and proportion of low and high-intensity activity for each individual player game file. Linear mixed models were used to quantify the magnitude of differences in each of these variables between positional groups.

**Results:**

Overall, across all positions, 33% of game play is spent completing low-intensity activity and 67% of high-intensity activity. There were minimal differences between positional groups in the mean, standard deviation and proportion of low and high-intensity activity, with total volume reflective of time spent on the field.

**Discussion:**

The lack of differences between positional groups suggests that accelerometers quantify both the running and contact that occurs within rugby league competition. As such, accelerometers may provide a measure of total high and low-intensity work in a contact based running sports, providing alternative and/or additional information to running metrics derived from global positioning systems.

## Introduction

Athletes engaged in team sports are subjected to a range of external forces impacting their bodies (1). In sport science, the term “load” refers to the stimuli that athletes encounter and respond to during training and competition (2). It is typically categorized as either internal or external load, and monitoring these variables is standard practice in team sport populations. Further, there is continued research and development into the methods used to quantify “load” in team sports. Global navigation satellite systems (GNSS) have emerged as a tool to quantify the running demands inherent in team sports. Moreover, the integration of inertial measurement units (IMUs) within GNSS provides a nuanced approach to quantifying the frequency of contact events (3), and stride parameters (4). The IMU unit typically consists of a GNSS unit, tri-axial accelerometers, gyroscopes and magnetometers (5). From these components, metrics such as the summation of the triaxial accelerations have gained prominence as a method for measuring the workload experienced by athletes (6). These triaxial measurement methods collectively contribute to an understanding of the physical load on athletes, offering insights to optimize training and tailor individual player training programs.

Rugby league involves a multifaceted array of sport-specific skills, prompting extensive research to quantify the physical exertion undertaken by players. This research (6, 7) has predominately used GNSS, extracting variables such as distance covered, mean speed, acceleration, high-speed distance, and counts of accelerations surpassing 2 m·s^−2^. Further, Delaney et al. (7) emphasized the significance of quantifying acceleration in rugby league and other team sports such as rugby union, AFL and soccer, attributing this importance to the stop-and-start nature inherent in the running activity within these sports. In addition to speed and acceleration variables, there has been methods such as metabolic power that amalgamate the load from both speed and acceleration (8). Nevertheless, it is essential to acknowledge that these GNSS metrics primarily capture the running demands of team sports, offering a limited perspective on the overall physical workload experienced within high contact sports such as rugby league.

For a comprehensive measure of athlete load, IMUs have been integrated into team sports research to account for both running and contact forces in team sports. “PlayerLoad™”, derived from triaxial accelerometers, has been extensively studied in Australian Football League (9), demonstrating a strong relationship with running volume. Within rugby league the research has primarily focused on the total PlayerLoad™ accumulation within games or training drills (10). More recent accelerometer advancements allow sport-specific movement detection, such as contact events, by setting a threshold that registers an impact when the total acceleration exceeds a set value (e.g., 5 G) (10). Moreover, accelerometer data has been used to quantify the peak impact occurring in tackles (11), although it was suggested that high sample rates (i.e., 1,000 Hz) may be required to quantify these short duration explosive movements. As such, triaxial accelerometers, offering higher sample rates than GPS, could show promise in quantifying the volume of work performed by players across both running and physical contact domains (12). Despite their potential, there is a dearth of literature applying accelerometers to quantify rugby league match play demands. While PlayerLoad™ effectively quantifies total load (12, 13), it lacks intensity details, indicating a breakdown of how PlayerLoad™ is accumulated. A player or position may have a greater value for high-intensity accelerometer data, however it needs to be contextualized to their total load. Unlike GNSS data, accelerometers have the capacity to capture tackle counts and dynamic movement patterns, providing greater understanding of workload intensity than PlayerLoad™ alone (13). Further research is warranted to explore the full potential of accelerometers in assessing rugby league match play loading across the intensity spectrum.

A study by Marutani et al. (14) was conducted that quantified the distribution of accelerometer data in official tennis matches. The proposed method involved assessing the external physical load by analysing the histogram of the “acceleration index” captured through wearable sensors. The acceleration index was calculated by summing the three accelerometer signals (x, y, z), subtracting the influence of gravity, and removing periods of rest (5 s of less than 0.3 G). This combined tri-axial accelerometer data demonstrated a bi-model distribution, suggesting work was accumulated at low and high-intensity (14). Subsequently, a gaussian mixture model was applied to the acceleration index distribution resulting in the mean, standard deviation, and proportion of low and high-intensity work. This extraction of features from the histogram-based analysis of the acceleration-derived signal offers new perspectives on monitoring the external physical load in diverse sporting activities. Indeed, by assessing the distribution of high and low intensity the acceleration index was able to accurately (93%) categorise collegiate and middle aged tennis players (14). Therefore, the purpose of the study is to quantify the distribution of low and high-intensity activity from accelerometer data in elite rugby league competition using similar methodology. Specifically, this study aims to determine the ability of accelerometers for measuring the volume and intensity of work in rugby league competition.

## Methods

The research is an observational study whereby accelerometer data is collected during rugby league match play. There were no adjustments to the athletes' match play routine and the data were collected, analyzed and interpreted as per the clubs standard monitoring processes. The study included 35 participants who were professional National Rugby League (NRL) players (age: 26.0 ± 3.4 years; height 187.8 ± 6.4 cm; weight 98.7 ± 9.7 kg). The participants were part of the male first grade squad and competed in the 2023 NRL competition. All rugby league positions were represented in the study including, middle forwards (*n* = 9), hookers (*n* = 3), edge forwards (*n* = 5), halves (*n* = 5), outside backs (*n* = 10) and fullbacks (*n* = 3). All participation was voluntary, with written consent provided by participants and ethic approval was provided by the Australian Catholic University Human Research Ethics Committee (2023-3121E).

There were no additional requirements of the athletes outside their standard in-season routine. Athletes wore a Catapult unit (Vector S7, firmware; 8.1, Catapult Sports, Victoria, Australia), secured in a playing jersey pouch, positioned at the posterior upper trunk, between the scapulae. The Catapult unit was fitted, turned on and monitored by the sports science staff of the respective club. This was a training load monitoring strategy utilized by the rugby league club at the time of undertaking the study, therefore the athletes are familiar with the devices and thus this study did not disrupt any current routines. The three-dimensional acceleration signals recorded at 100 Hz across the entire 2023 NRL season (27 matches) for all 35 athletes.

**Figure 1:**
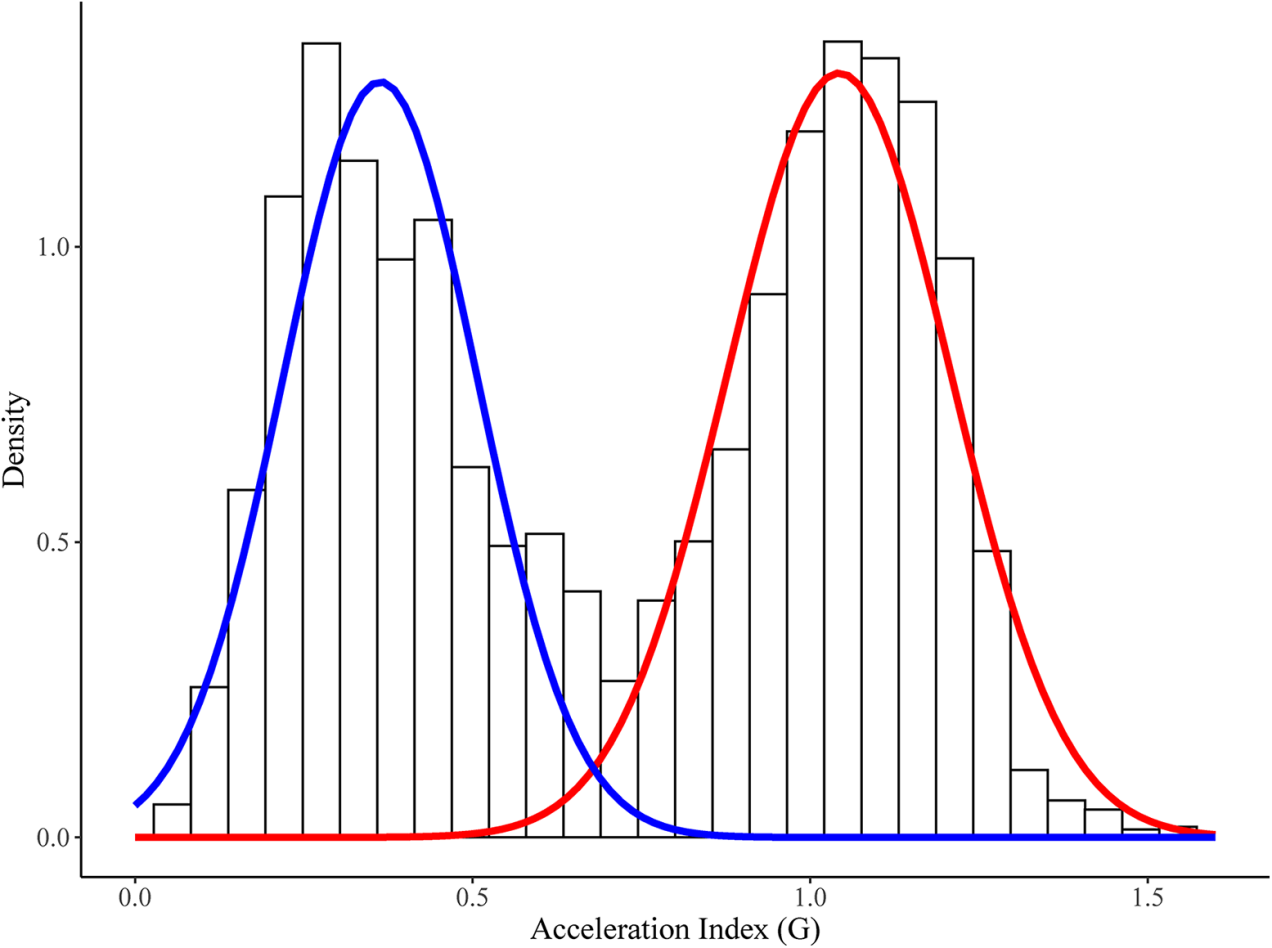
Example density plot of the rugby league match play accelerometer data with fitted bimodal Gaussian mixture model. In this example, the low-intensity distribution had a mean of 0.363 G, a standard deviation of 0.144 G and a proportion of 0.463. The high-intensity distribution had a mean of 1.043 G, a standard deviation of 0.166 G, and a proportion of 0.537.

The 100 Hz triaxial accelerometer data was exported into individual comma separated value (.csv) files. Individual game data files were then manually processed using RStudio (RStudio v. 1.4.1106, RStudio, Boston, MA) following methods previously described (14). Briefly, individual accelerometer axis (i.e., X, Y, Z) were firstly filtered using a 2nd order Butterworth band-pass filter (0.5–20 Hz) to remove the low and high frequency noise and gravity influences from the acceleration signals. The three-accelerometer axis were when combined into an overall accelerometer signal by finding the square root of the summed squared axis. The summated acceleration signal was then smoothed over a 1 s duration using a moving average. To determine when activity was occurring a secondary acceleration signal was established using a 5 s (500 datapoint) moving average. Where values for this signal were below 0.3 G, the data were removed from the dataset. The histogram of the acceleration index produced a clear bimodal distribution, which indicated high and low-intensity peaks (see Figure 1). Using the “Mclust” function within the “mclust” library (15) a Gaussian mixture model was then fitted to the two clusters of data (computation time under one second), resulting in a mean, standard deviation, and proportion of each of the distributions for each player for each game. As such, there are no “thresholds” for determining what is high and low-intensity, rather the distribution of the acceleration index determines what is high and low-intensity and the mean, standard deviation and proportion of high and low acceleration index values quantified the total work undertaken by the players. Similarly to previous work on PlayerLoad™, a summation of the acceleration index was also calculated in arbitrary units for each individual player game file.

Linear mixed models were used to examine the magnitude of difference between positional groups for each of the variables extracted from the Gaussian mixture model. The least squares mean test was used to obtain positional comparisons, and the resulting standard deviations (SDs) and mean differences in accelerometer derived values between positional groups were used to establish standardized effect sizes (ES) and 90% confidence limits (CL). Standardized ESs were described using the magnitudes; 0.20 trivial; 0.21–0.60 small; 0.61–1.20, moderate; 1.21–2.0 large and 2.01 very large (14). Differences were deemed to be real if they were 75% greater than the smallest worthwhile difference (calculated as 0.2× the between- athlete SD). Further, for each athlete, a within athlete z-score for each variable is established. Within player *Z*-scores for both volume and intensity were also established to assess the interaction between these two variables for individual players.

## Results

Table 1 provides a summary of the high and low-intensity accelerometer index variables extracted from the data collected during rugby league competition by positional group. When examining the volume of low and high-intensity activity the middle forwards had a lower volume of low-intensity activity compared to hookers (ES: −3.5; 90% CI: −6.1—−1.0) and outside backs (−1.4; −3.0–0.1). Middle forwards also had a lower volume of high-intensity activity compared to halves (−2.0; −3.7–0.3). This resulted in a lower total acceleration volume in middle forwards compared to halves (−1.9; −3.7–0.2) and hookers (−2.9; −5.6–0.2). The mean of high-intensity activity in outside backs was substantially higher than middle forwards (1.7; 0.2–3.2) and halves (1.8; 0.8–2.8). All other position comparisons across variables were not substantial.

**Table 1:** Accelerometer index variables (mean ± SD) extracted from the Gaussian mixture model by positional group.

Acceleration index	Middle forward	Hooker	Edge forward	Halves	Outside back	Fullback
Volume (AU)						
Low	33.8 ± 11.0^b^,^d^	66.9 ± 26.9	45.8 ± 11.1	57.8 ± 16.8	55.7 ± 18.2	61.4 ± 9.0
High	76.4 ± 29.2^a^	117.1 ± 48.3	108.5 ± 31.8	130.2 ± 21.7	107.8 ± 28.2	124.6 ± 18.8
Total	110.3 ± 38.6^a^,^b^	184.0 ± 73.1	154.3 ± 41.6	188.1 ± 33.0	163.5 ± 43.5	186.0 ± 26.5
Mean (G)						
Low	0.32 ± 0.03	0.35 ± 0.06	0.32 ± 0.03	0.30 ± 0.03	0.31 ± 0.02	0.31 ± 0.01
High	0.92 ± 0.06	0.97 ± 0.04	0.91 ± 0.03	0.89 ± 0.08	0.97 ± 0.04^a^,^c^	0.96 ± 0.02
SD (G)						
Low	0.09 ± 0.02	0.10 ± 0.05	0.08 ± 0.02	0.08 ± 0.02	0.08 ± 0.01	0.09 ± 0.01
High	0.29 ± 0.04	0.26 ± 0.07	0.30 ± 0.04	0.27 ± 0.04	0.30 ± 0.03	0.25 ± 0.02
Proportion						
High	0.68 ± 0.07	0.63 ± 0.07	0.69 ± 0.08	0.70 ± 0.06	0.66 ± 0.06	0.67 ± 0.2

^a^ Substantially different to halves.

^b^ Substantially different to hooker.

^c^ Substantially different to middle forwards.

^d^ Substantially different to outside backs.

**Figure 2:**
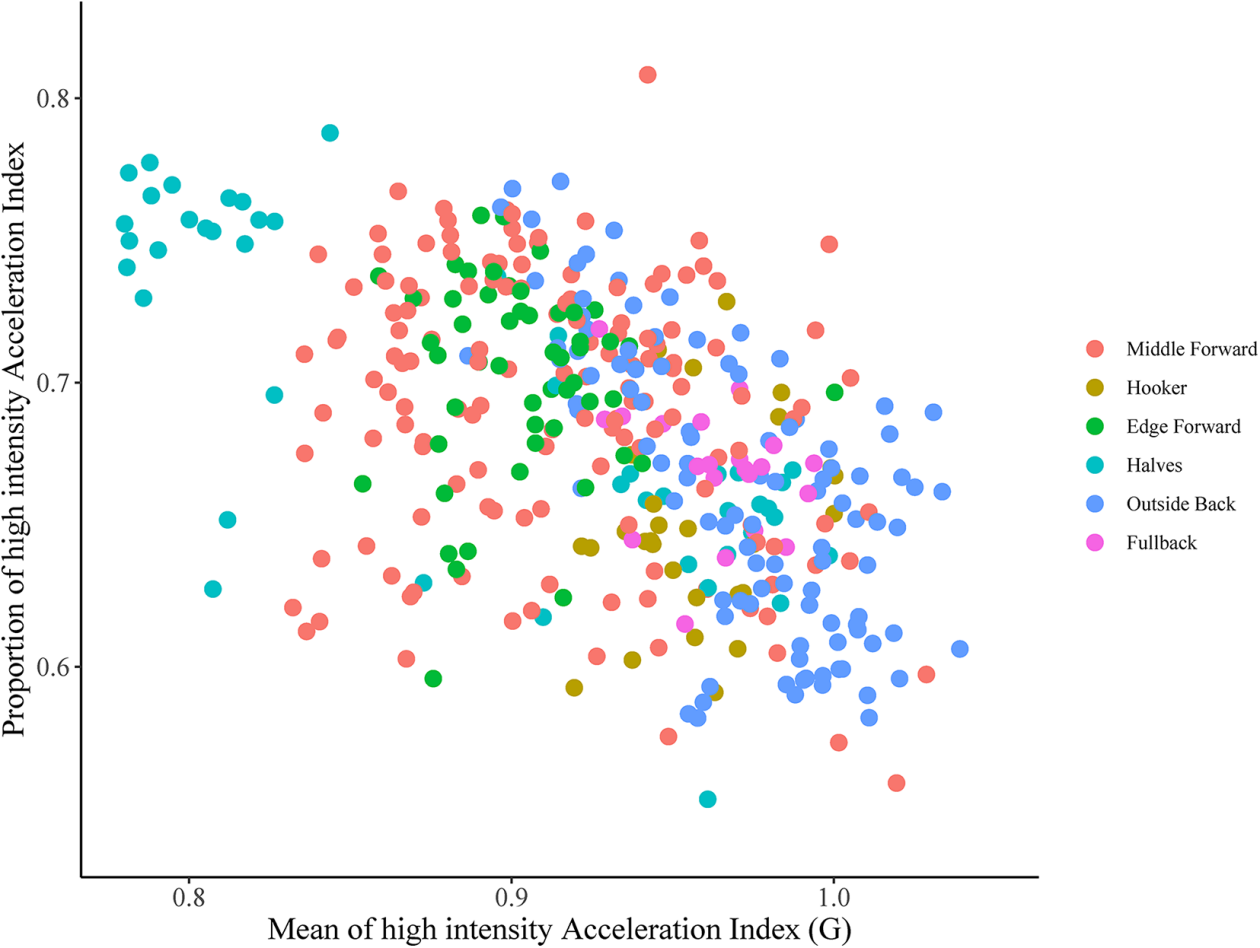
The mean of the high-intensity accelerometer index (X axis) expressed relative to the proportion of high-intensity acceleration index (Y axis).

**Figure 3:**
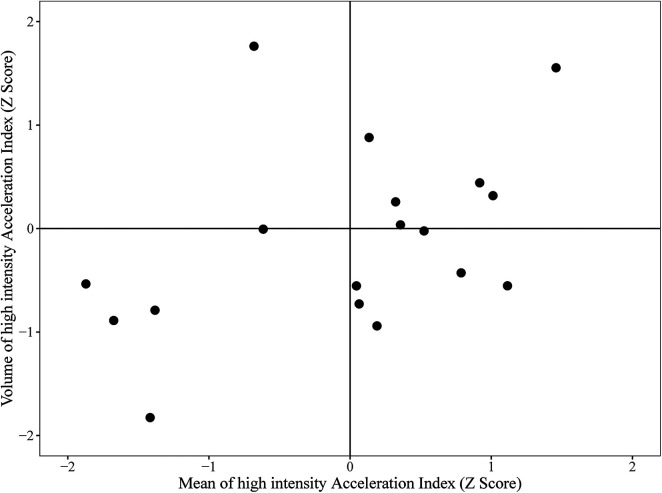
The *x* axis is the *z*-score of the volume of high-intensity accelerometer data from each game. The *y* axis represents the individual players z-score of the mean high-intensity accelerometer data from each game. Data are presented as within-athlete *Z*-scores (represents how each match compares to all other matches).

Figure 2 demonstrates the mean and proportion of high-intensity acceleration index data for each athlete in each match, by their respective position played. Figure 3 is an example of an individual athletes *Z* score for the high-intensity acceleration index mean and volume plotted for each match of the season. Subsequently, the top right quadrant represents games where the athlete registered a higher acceleration index for high-intensity activity, coupled with a higher volume of high-intensity for the acceleration index, suggesting a highly demanding game. Conversely, the lower left quadrant represents the opposite, where the athlete had a lower mean value for the high-intensity acceleration index coupled with a lower volume of high-intensity acceleration index compared to their usual match play values. This figure provides an example of how this analysis can potentially be used to quantify volume and intensity of work performed in rugby league competition.

## Discussion

This research provides a novel analysis of accelerometer data collected during rugby league match play, marking the first instance of such an approach to measure external load within a team sport. While previous analyses predominantly center on GNSS analysis of speed and acceleration, this study introduces a novel method using IMU-derived accelerometer data. Specifically, the analysis involved quantifying the distribution of accelerometer data of an elite rugby league competition and establishing the accelerometer index. For individual player game data, the summed accelerometer values (i.e., “Accelerometer Index”) demonstrated a bimodal distribution suggesting significant volumes of low and high-intensity activity. By applying a Gaussian mixture model, information from the two distributions, (i.e., mean, standard deviation, and proportion) which represent high and low-intensity activity was extracted (16). Lastly, this study used within-athlete *Z*-scores as a method of presenting the interplay between the volume and intensity of acceleration of individual matches, demonstrating use as a practical tool of describing the acceleration index of each match. Overall, this study provides a greater understanding of the external load an athlete has undertaken when used in conjunction with traditional GNSS analysis of speed and acceleration.

Using a Gaussian mixture model, an array of variables was extracted from the accelerometer data to comprehensively examine the external load experienced by athletes. Notably, these variables encompassed the mean values and proportion of both low and high-intensity activities. Low-intensity activities serve a strategic purpose, enabling athletes to maintain optimal positioning in response to dynamic game scenarios, and conserve energy for pivotal moments (17). These activities are crucial for effective gameplay, as they allow athletes to sustain performance throughout the match, particularly in scenarios that demand rapid tactical adjustments without significant energy expenditure (18). Conversely, high-intensity activities are characterized by rapid and forceful alterations in speed or movement, reflecting the dynamic nature of the sport. High-intensity activities typically include sprints, sudden directional changes, and explosive bursts of speed, which are essential for breaking through defensive lines, making tackles, and executing strategic plays. Across positions, variations in total acceleration volume were primarily attributed to differences in on-field duration, with middle forwards typically engaging in rotational play, while other positions remain on field for the duration of the game (80-min). Middle forwards, due to their rotational nature, experience fluctuating periods of intense activity interspersed with rest, allowing them to recover and re-engage in high-intensity play. In contrast, positions such as fullbacks and outside backs often sustain prolonged periods of moderate to high-intensity activity without the benefit of regular rest intervals. Surprisingly, the mean acceleration index for high and low-intensity activity demonstrated minimal differences across positions, contrary to previous observations suggesting fullbacks endure the greatest workload (17). This discrepancy underscores the limitations of GNSS-based analyses, which fail to capture the physical demands associated with the contact elements of the game. Fullbacks, traditionally believed to experience higher workloads, may not exhibit significantly higher mean intensities due to the frequent, yet less intense, activities required to maintain their strategic positioning and cover large areas of the field (19, 20). Consequently, the current methodology affords a nuanced understanding of player exertion in rugby league. By capturing both the intensity and context of physical activities, this approach provides novel insights into the multifaceted nature of performance and the varied physical demands across different positions.

Across positions, the accelerometer index suggested approximately 33% of the activity within rugby league match play was characterized as low-intensity, while 67% was classified as high-intensity. The Gaussian mixture model allowed for the proportion, mean, and SD of the accelerometer index to be extracted. Such information has the potential to inform practitioners regarding the global (i.e., running and contact) demands of competition. For example, despite research in Australian Football showing a strong relationship between PlayerLoad and Total Distance (9), perhaps variations in this relationship will be observed in higher contact sports such as the Rugby codes where contact is a significant contributor to the overall “load” experienced by the players. This could provide insights in training regimen development, injury mitigation strategies, and recovery protocols. Further, by understanding the proportion of low vs. high-intensity activities, coaches and sports scientists can tailor training programs to better simulate match conditions, ensuring players are adequately prepared for the physical demands of competition (21). Notably, while a player or position may register a high mean value for high-intensity accelerometer data, it's imperative to contextualize this within the broader spectrum of their workload. Unlike GNSS data, which primarily quantifies running activities, accelerometer data offers insights into additional components of the game, such as tackles, jumps, and changes in direction. This study illustrates the utility of accelerometers in scrutinizing the intensity and volume of individual player performances over the course of a season. This comprehensive data collection allows for a more detailed analysis of player workload. Moreover, by expressing individual game data as *z*-scores, we highlighted how it can be established if a player had a higher demanding game based on intensity and volume, further enhancing our understanding of player exertion dynamics. This approach not only identifies matches that are outliers in terms of physical demand but also assists in planning recovery strategies and future training sessions tailored to the specific needs of the player (21). Being able to identify taxing matches, coaching staff can prioritise rest and recovery, adjust training loads, and implement targeted conditioning programs to optimize player health and performance throughout the season.

This paper does not provide a direct comparison between GNSS and accelerometers. Instead, it offers insights into the use of accelerometers for quantifying the volume and intensity of contact sports, specifically rugby league. Certain facets of contact sports are arguably overlooked when solely relying on GNSS technology, as it often fails to discern intricate movements such as jumping, falling, tackling, and other critical skills. The novel analysis of accelerometer data presented in this study enables a more comprehensive evaluation of the total workload undertaken by athletes. Athlete monitoring devices play a pivotal role in collecting and analyzing the activities undertaken by team sport athletes, facilitating coaches and high-performance personnel in monitoring training loads and optimizing player recovery. Integrating accelerometer data analysis with GNSS information yields a more holistic depiction of athletes' external loads (21). While the findings offer an alternative avenue for analyzing total external load in contact sports, it's imperative to acknowledge inherent limitations. Accelerometer data generation entails the accumulation of extensive raw data necessitating meticulous processing and interpretation. This process can be complex and time-consuming, requiring proficiency in data analytics. For some teams or organizations, particularly those with limited resources, this may pose significant challenges. Ensuring that staff are proficient in data analysis or have access to advanced analytical tools is essential for effectively utilizing accelerometer data (22).

The integration of accelerometer data with GNSS technology provides a more comprehensive and accurate assessment of external player workload in rugby league. This approach enhances our understanding of the physical demands of the sport, aiding in the development of tailored training programs and effective recovery protocols. However, it is crucial to address the challenges associated with data processing and analysis to fully leverage the benefits of this advanced monitoring technology. This paper can provide not only rugby league high performance staff, but all contact sport staff and professionals with an alternate insight to player load management and how it can be displayed and interpreted differently to the commonly used GNSS data collected. More specifically, the methodology within this study demonstrates the capabilities of measuring the external load encountered within contact sports, opposed to solely distance and speed variables. Overall, accelerometers are a non-invasive method to quantify sporting demands of athletes.

This article adopted the use of the Gaussian mixture modelling to determine players means, standard deviations and proportions of acceleration signals from wearable microsensors. This paper utilized methodology previously introduced to measure external load in tennis (17), but applies it to contact sport, where there is a clear deficiency in measuring external load in its entirety. Briefly, the data processing involved some bascis filtering of the raw individual accelerometer axis data, followed by summation of these axis. Following this the individual axis are summated and periods of activity identified. Using the “mclust” library in R allows the fitting of a Guassian mixture model to identify clusters of low and high intensity and these are summarised by the mean, standard deviation and proportion of low and high intensity activity. These variables can be examined within individuals to further contextualise the volume of intensity of activity performed. There are several limitations to this study, in particular the data was only collected on players from one club within the NRL competition and this resulted in at limited sample size in several positions, most notably the hooker and fullback positions. Further, this investigation used a single microtechnology brand (i.e., Catapult Vector S7). Despite analysing the raw tri-axial accelerometer output there may be subtle filtering and data processing techniques employed by the manufacturer prior to the raw data export. This processing could vary between different providers and would therefore influence the final result achieved. Regardless, high performance staff should have the confidence in utilizing accelerometers to measure player load to improve preparation, training, performance and recovery of athletes. Although further research is required to determine their validity in measuring certain variables from accelerometers this study has provided an alternative method to purely examining GNSS data and/or the total “PlayerLoad™” experienced by the player.

## Data Availability

The raw data supporting the conclusions of this article will be made available by the authors, without undue reservation.
